# Zinc attenuates ecstasy-induced apoptosis through downregulation of caspase-3 in cultured TM3 cells: An experimental study

**DOI:** 10.18502/ijrm.v13i9.7672

**Published:** 2020-09-20

**Authors:** Marziyeh Lozeie, Morteza Bagheri, Isa Abdi Rad, Nadia Hossein-Zadeh, Mahdyieh Nasir-Zadeh

**Affiliations:** ^1^Islamic Azad University, Tabriz Complex, Tabriz, Iran.; ^2^Cellular and Molecular Research Center, Cellular and Molecular Medicine Institute, Urmia University of Medical Sciences, Urmia, Iran.

**Keywords:** Zinc, MDMA, Apoptosis, TM3 cells

## Abstract

**Background:**

3, 4-Methylenedioxymethamphetamine (MDMA) is commonly known as the most famous amphetamine derivative.

**Objective:**

To evaluate the influence of zinc on MDMA-induced apoptosis and caspase- 3 gene expression in Leydig cell line (TM3).

**Materials and Methods:**

Leydig cells were studied in differenet treatment groups regarding MDMA (0, 0.5, 1, 3, 5 mM) and zinc (0, 4, 8, 16, 32 μM). By the way, the effective concentration was determined to be 5 mM for MDMA and 8 μM for zinc. Then, TM3 cells were cultured in free medium as control (group I), medium containing MDMA (5 mM) (group II), zinc (8 µM) (group III), and zinc (8 µM) prior to MDMA (5 mM) (group IV) as well as in an untreated group (control). Cell viability was assessed at different times after cell culture by MTT assay. The mRNA expression level of caspase-3 was analyzed using real-time quantitative polymerase chain reaction.

**Results:**

The cellular viability was significantly reduced in TM3 cells after 24 hr and 48 hr exposure time regarding different concentrations of MDMA as well as high concentration of zinc (16 and 32 μM). Cell viability was increased in the group that received zinc (8 µM) before addition of MDMA (5 mM) compared to the control and MDMA groups. The mean ± SE of fold was 22.40 ± 7.5, 0.06 ± 0.02, and 0.009 ± 0.003 in MDMA, zinc, and zinc + MDMA groups, respectively. The mean of caspase-3 mRNA level was significantly increased in the MDMA-treated group (5 mM), while the relative expression of caspase-3 gene was significantly decreased in the zinc (8 µM) + MDMA (5 mM) group compared with the MDMA (5 mM) group (p = 0.001).

**Conclusion:**

Dietary intake of zinc has a protective effect against MDMA consumption in mouse.

## 1. Introduction

3, 4-Methylenedioxymethamphetamine (MDMA; “Ecstasy”) is commonly known as the most famous amphetamine derivative. The toxic effects of ecstasy include: hyperthermia, rhabdomyolysis, coagulopathy, or bleeding abnormalities, and acute renal failure (1, 2). MDMA increases the secretion of three neurotransmitters of serotonin, dopamine, and norepinephrine from the axon terminals in the synapse. Serotonin influences mood, appetite, and sleep. It also triggers the secretion of the oxytocin and vasopressin hormones that control social behaviors (3). Ecstasy affects the endocrine system and the hypothalamic-pituitary-thyroid axis, leading to an increase in body temperature (4). Ecstasy with effect on the hypothalamic-pituitary-adrenal axis leads to an increase in the secretion of adrenocorticotropin hormone and cortisol. As a result, the level of free radicals is increased, resulting in ecstasy-induced neurotoxic effects (5, 6).

Chronic use of ecstasy has direct effects on the reproductive system. Ecstasy causes damage to testicular tissue and reduces spermatic production by increasing oxidative stress and apoptosis (7, 8). The mechanism(s) of ecstasy-induced infertility is not fully understood (9). Recent studies have shown that the production of excess amounts of free radicals can lead to serious damage to sperm (10-13). It has been demonstrated that oxidative stress influences the human fertility; and most specialists do not assess their patients regarding oxidative stress (14). In males, the interstitial, or Leydig, cells are placed in the connective tissue nearby the sperm-producing tubules of the testicles (gonads), and produce testosterone (15). The synthesis of androgens is controlled by hypothalamus-pituitary-testicular axis via pituitary secretion of LH. Leydig cells have LH receptors and maintain spermatogenesis through the hypothalamic-pituitary-dependent feedback mechanism (15). The effect of ecstasy on Leydig cells and testicular tissue has not been broadly investigated. At least from the outlook of apoptosis in Leydig cells, there is no report. It has been indicated that ecstasy is toxic to Leydig cells, and leads to the decreased level of normal cells and the increased level of DNA damage in ecstasy-treated Leydig cells (16).

The effect of ecstasy on male reproductive system is may be due to the stimulation of oxidative stress (17). Zinc has a protective influence on human reproductive system by reducing free radicals (18). Zinc deficiency can therefore adversely affect the function of zinc-dependent proteins in biological systems (19). Moreover, the growing bodies of evidences suggest that zinc deficiency increases the secretion and production of inflammatory cytokines as well as the phenomenon of oxidative stress (19-22). In men, antioxidants prevent the harmful effects of oxidative free radicals on spermatogenesis and sperm health and reduce the amount of testicular oxidative stress (23).

This study was designed to investigate the impact of MDMA, zinc and zinc + ecstasy on TM3 cells as well as the mRNA level of caspase-3 in the treated and control groups.

## 2. Materials and Methods

### Cell culture protocol

In the experimental study, we used Leydig Mouse Cells (TM3) obtained from the Pasteur Institute of Iran. The cells were cultured in DMEM/F12 (ATOCEL, Austria) enriched with 10% FBS (ATOCEL, Austria) and 1% penicillin-streptomycin (ATOCEL, Austria). Following the initial cell-plating density, culture flasks were incubated in humidified 5% CO${}_{2}$ incubator at 37°C. Cells between passages 3 and 6 were used for various analyses. For cell passage, TM3 cells were detached by using 0.25% Trypsin-EDTA solution (ATOCEL, Austria).

### MTT assay

TM3 cells were cultured at a density of 5 × 103 cells per well in a 96-well culture plate (SPL, Korea) and were kept at 37°C under 5% CO${}_{2}$. The minimal effective concentration of ecstasy and zinc were evaluated regarding different concentrations of ecstasy and zinc. In this case, after a 24 hr stabilization period, cells were treated with ecstasy (0, 0.5, 1, 3, and 5 mM) for 24 hr and 48 hr. Also, to determine the effective concentration of zinc, TM3 cells were treated with 0, 4, 8, 16, and 32 µM zinc sulfate (sigma, USA) for 24 hr and 48 hr in a final volume of 100 µl. The protective effect of zinc on ecstasy induced apoptosis was tested by pretreatment with zinc. A pretreatment for 24 hour was performed with 8 µM zinc prior to ecstasy (5 mM) exposure. At the end of the incubation time, the supernatant media were discarded and replaced by 100 μl PBS containing 1 mg/ml MTT (3-(4, 5-Dimethylthiazol-2-yl)-2, 5-diphenyltetrazolium bromide, Sigma).

Next, after 4 hr, 100 μl dimethyl sulfoxide (Merck, Germany) was added and kept for 15 min at room temperatture. Using a microplate reader (Bio-Tek, USA), each sample's absorbance was measured at 545-630 nm. The experiments were repeated in triplicate and results expressed as % of non-treated control. Based on the results from cell survival assay, the effective concentration of ecstasy and zinc was determined. Then, TM3 cells were cultured in four groups and examined. The TM3 cells were cultured in free medium as control (group I), and medium containing ecstasy (5 mM) (group II), medium containing zinc (8 µM) (group III), and medium containing zinc (8 µM) prior to MDMA administration (5 mM) (group IV). In this regard, the TM3 cells were pre-treated with zinc (8 µM) for 24 hr before incubation with ecstasy (5 mM). The MMT assay was performed for evaluating the cell survival rate in the tested groups.

### RNA extraction, cDNA synthesis, and real-time PCR (RT-PCR)

10${}^{5}$ TM3 cells/wells were cultured in 6-well plates for 24 hr. The cells were examined in four groups including ecstasy (5 mM), zinc (8 µM), pre-treatment, and control. After 24 hr, while the well of pretreatment group was treated with zinc, the other wells were treated with free medium for 24 hr. The medium was changed with fresh medium. On the next day, the medium was aspirated from each well and exchanged with an ecstasy containing medium for wells of pre-treatment and ecstasy groups, with a zinc-containing medium for zinc group and with free medium for control group. After 24 hr of last treatment, the cells trypsinized and the cells pellet was used to extract RNA after centrifugation at 3000 rpm for 10 min. RNA extraction was carried out using the RNX Plus Solution Kit (SinaClon) (Catalog Number: RN7713C). Two sets of forward and reverse primers were used for the target gene (Caspase-3) (GCA GCT TTG TGT GTG TGA TTC and AGT TTC GGC TTT CCA GTC AG) and reference gene (beta-actin) (TAG GCG GAC TGT TAC TGA GC and GCT CCA ACC AAC TGC TGTC). The PCR program included 94°C for 30 sec; 60°C for 40 sec; and 72°C for 50 sec (35 cycles) (24). The RNA concentration of all specimens was confirmed and the synthesis of cDNA was done through the following compounds: total RNA was used to generate single-stranded cDNA with 2-step RT-PCR kit (Thermo Scientific RevertAid First Strand cDNA Synthesis Kit #K1622). The cDNA synthesis of the samples was carried out in thermocycler for 60 min, according to the program presented in the product certificate at 25°C for 5 min and 42°C for 60 min. Then, the synthesized cDNA was used in this step to perform real-time PCR. The RT-PCR was done using the Applied Biosystems StepOne RT-PCR System.

### Ethical consideration

This study has been approved by the research ethics committee of the Urmia University of Medical Sciences (IR.UMSU.REC.1397.448).

### Statistical analysis

The RT-PCR results were analyzed using the 2-ΔΔCT method. To analysis the data, the Statistical Package for the Social Sciences, version 20, SPSS Inc, Chicago, Illinois, USA (SPSS) software was used. The statistically significant data was determined using the one-way analysis of ANOVA, followed by Tukey's test. Additionally, to determine the significance of the results, the value of P was considered as 0.05.

## 3. Results

**Figure 1:**
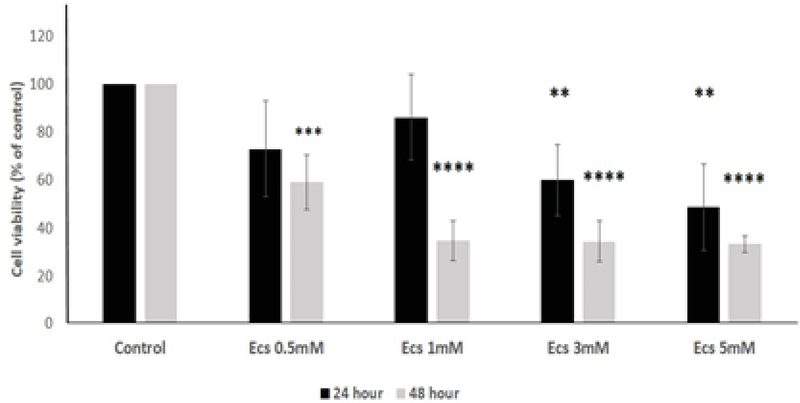
The effect of ecstasy on TM3 cell viability. Comparison of the effects of different concentrations of ecstasy on TM3 cell viability following 24-hr and 48-hr exposure period. Data are expressed as the Mean ± SD. *P < 0.05; **P < 0.01; ***P < 0.001; ****P < 0.0001.

**Figure 2:**
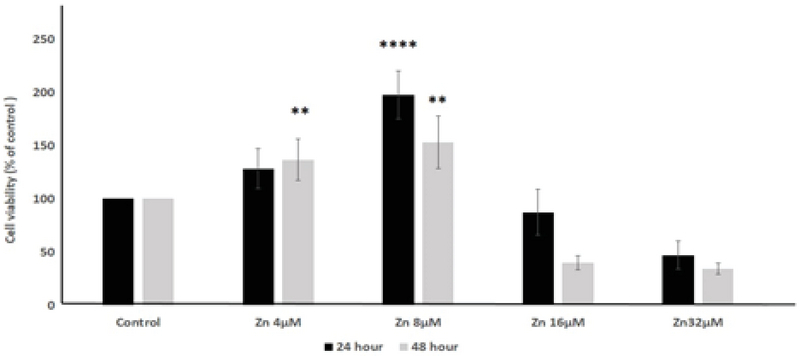
The effect of Zinc on TM3 cell viability following 24-hr and 48-hr exposure period. Cellular viability increased at low concentrations (4 and 8 µM) but decreased in the case of high concentrations (16 and 32 µM). Data are expressed as the Mean ± SD. *P < 0.05; **P < 0.01; ***P < 0.001;****P < 0.0001.

**Figure 3:**
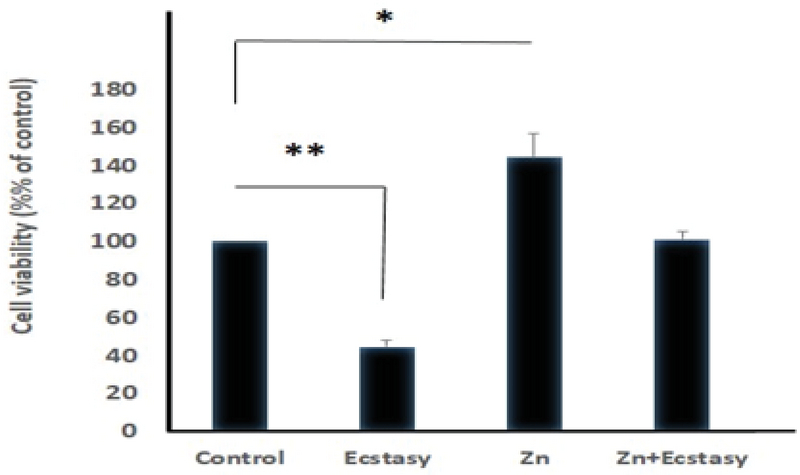
Comparison of the effects of different treatments on TM3 cell viability. The experimental groups were assigned to treatment conditions that included ecstasy (5 Mm) (group II), Zinc (8 µM) (group III), pretreatment (group IV), and control (group I) groups. In the pretreatment group, the cells were pretreated with zinc (8 µM), for 24 hr, before ecstasy (5 mM) exposure. Data are expressed as Mean ± SD. *P < 0.05; **P < 0.01.

**Figure 4:**
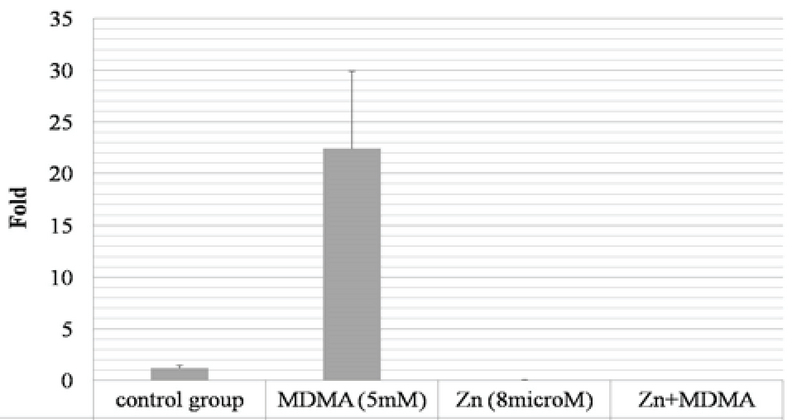
The relative amounts of caspase-3 gene expression were detected by RT-PCR in tested groups including control (group I), MDMA (5 mM) (group II), zinc (8 μM) (group III), and zinc + MDMA (group IV) groups. The Mean (± SE) of fold was 22.40 (±7.5), 0.06 (±0.02), and 0.009 (±0.003) in group II, III, and IV, respectively. Data represent the mRNA levels versus the control group. *P = 0.001; **P = 0.993; and ***P = 0.992 compared with the control group (one-way ANOVA and Dunnett *t* tests treat).

**Figure 5:**
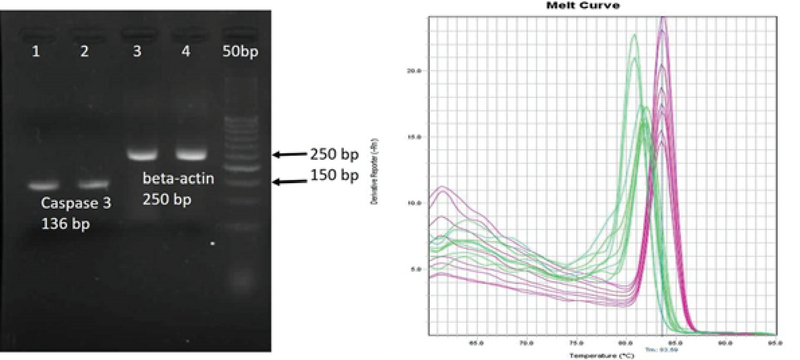
L: Representative gel image of RT-PCR products in TM3 cells; R: Melting curve analysis of caspase-3 and beta-actin primers which showed a single peak for each and high specificity.

Cell viability was significantly reduced in the TM3 cells treated with different concentrations of ecstasy (0, 0.5, 1, 3, 5mM) for 24 hr and 48 hr. In both 24 hr and 48 hr exposure time, ecstasy decreased the cell viability in a dose/time-dependent manner with IC50 values of 5 mM for 24 hr and 1 mM for 48 hr exposure (Figures 1). In the case of zinc, our study indicated that cell viability became increased in comparison to the control in only lower concentration of zinc in 24 hr and 48 hr exposure time. However, cell viability was decreased regarding high concentrations of zinc (Figures 2). A concentration of 5 mM for ecstasy and 8 µM for zinc was found as effective concentrations (Figure 3). In group IV, cell viability became increased in comparison to groups I and II. Morever, in this study, ampliﬁcation efficiencies were set at 90-105%. Our findings showed that the mean (± SE) of fold was 22.40 ± 7.5, 0.06 ± 0.02, and 0.009 ± 0.003 in group II, III, and IV, respectively. The mean of caspase-3 mRNA level (fold) was significantly increased by treatment with ecstasy in group II. The relative expression of caspase-3 gene was significantly decreased in the zinc + ecstasy group (group IV) compared with the ecstasy (5 mM) group (p = 0.001) (Figure 4). These results indicated that zinc has the inhibiting effect on apoptosis through caspase-3-mediated pathway in the TM3 cells. Figure 5 shows gel image and melting curve analysis of tested gene RT-PCR products.

## 4. Discussion

The present research studies the antioxidant activity of zinc. The foremost finding of the present investigation was the attenuation of caspase 3 gene expression by zinc following MDMA treatment. Pretreatment with zinc was protective against MDMA-induced apoptosis in TM3 cells. Our results are in agreement with the other studies and imply that zinc has protective and anti-apoptotic effects at low concentrations by inhibition of oxidative stress damage (16, 22). Several studies indicated that the consumption of ecstasy may result in damage of numerous organs such as the heart, liver, kidney, and central serotonergic (5-HT) systems, and death by unknown mechanisms (24-27). Montiel-Duarte and colleagues showed that exposure to ecstasy caused apoptosis of cultured rat liver cells (17). Also, Pourhassanali and colleagues showed that ethanol-induced toxicity in mouse Sertoli cells decreased via zinc pre-treatment (16).

It has been demonstrated that ecstasy raises DNA break in sperm and modifies testes tissue (7). Ecstasy induces apoptosis in wide range of cell lines and organs such as testes, liver, and brain (28). In testes, the cell death occurs by apoptosis via hazardous materials (ecstasy, ethanol, deprivation of intra-testicular testosterone and serum levels of gonadotrophins, Sertoli cell toxicants, chemotherapeutic drug, etc.) (29). All these facts warn a serious necessity to train people around the toxicities of ecstasy especially in early reproductive age. The biological systems are sensitive to oxidative stress (30). For that reason, external consumption of antioxidant is known as one of the most general curative strategies against hazardous materials. In this regard, numerous antioxidants have been investigated in relation to the oxidative stress (31).

It has been demonstrated that MDMA results in intracellular Ca2+ overflow, depolarization of mitochondrial membrane, reactive oxygen species (ROS) production, and activation of Caspase-9 (32). At lower concentrations, ROS has been allied to the stimulation of cell survival reactions, but in the case of higher concentrations, it activates apoptosis via activation of caspases-3, -8, and -9 (33). Caspase-dependent apoptosis has been studied in several human diseases including cancer, neurological disorders, cardiovascular disorders, autoimmune diseases, and male infertility. Apoptosis has a central role in spermatogenesis (34).

In spermatogenesis, a lot of the developing germ cells pass away through apoptosis before maturity (35). “The physiological cell apoptosis occurs during life, but increased germ cell apoptosis results from external disturbances" (36). According to these findings in the present study the protective effect of zinc against ecstasy induced- apoptosis seems to be related to its potent antioxidant properties. Maintaining physiological concentrations of zinc and its tight control by MTs in each cell of the body is necessary to avoid oxidative stress, since not only zinc deficiency but also zinc overload are pro-oxidant conditions (due to the inhibition of mitochondrial respiration and antioxidant enzymes) (37, 38). Therefore, the protective effect of zinc might be associated to its antioxidant effects. Our findings for the first time not only demonstrated that ecstasy has cytotoxic effect on the TM3 cells and induced apoptosis via over-expression of caspase-3, but also zinc inhibited ecstasy-induced testicular injuries.

## 5. Conclusion

It can be concluded that dietary intake of zinc has a protective effect against MDMA consumption. These data suggest a possible underlying molecular mechanism for MDMA to induce the apoptosis signaling pathway by upregulation, and also, pretreatment with zinc attenuated apoptosis by down-regulation of caspase-3 gene expression in TM3 cells.

##  Conflicts of Interest 

None.
